# 5′ and 3′ Untranslated Regions Strongly Enhance Performance of Geminiviral Replicons in *Nicotiana benthamiana* Leaves

**DOI:** 10.3389/fpls.2016.00200

**Published:** 2016-02-24

**Authors:** Andrew G. Diamos, Sun H. Rosenthal, Hugh S. Mason

**Affiliations:** Center for Infectious Diseases and Vaccinology, Biodesign Institute, and School of Life Sciences, Arizona State University, TempeAZ, USA

**Keywords:** geminivirus, *Nicotiana benthamiana*, 5′ untranslated regions, 3′ untranslated regions, monoclonal antibody, transient expression, virus-like particle

## Abstract

We previously reported a recombinant protein production system based on a geminivirus replicon that yields high levels of vaccine antigens and monoclonal antibodies in plants. The bean yellow dwarf virus (BeYDV) replicon generates massive amounts of DNA copies, which engage the plant transcription machinery. However, we noticed a disparity between transcript level and protein production, suggesting that mRNAs could be more efficiently utilized. In this study, we systematically evaluated genetic elements from human, viral, and plant sources for their potential to improve the BeYDV system. The tobacco extensin terminator enhanced transcript accumulation and protein production compared to other commonly used terminators, indicating that efficient transcript processing plays an important role in recombinant protein production. Evaluation of human-derived 5′ untranslated regions (UTRs) indicated that many provided high levels of protein production, supporting their cross-kingdom function. Among the viral 5′ UTRs tested, we found the greatest enhancement with the tobacco mosaic virus omega leader. An analysis of the 5′ UTRs from the *Arabidopsis thaliana* and *Nicotinana benthamiana* photosystem I K genes found that they were highly active when truncated to include only the near upstream region, providing a dramatic enhancement of transgene production that exceeded that of the tobacco mosaic virus omega leader. The tobacco Rb7 matrix attachment region inserted downstream from the gene of interest provided significant enhancement, which was correlated with a reduction in plant cell death. Evaluation of *Agrobacterium* strains found that EHA105 enhanced protein production and reduced cell death compared to LBA4301 and GV3101. We used these improvements to produce Norwalk virus capsid protein at >20% total soluble protein, corresponding to 1.8 mg/g leaf fresh weight, more than twice the highest level ever reported in a plant system. We also produced the monoclonal antibody rituximab at 1 mg/g leaf fresh weight.

## Introduction

Recombinant protein production systems have become an integral part of medicine, industry, and research. Biopharmaceutical proteins, including monoclonal antibodies, enzymes, growth factors, and other biologics, are the largest and fastest growing sector of all pharmaceuticals (Butler and Meneses-Acosta, 2012). Nearly all of these recombinant proteins are made with traditional bioreactors using mammalian, insect, or microbe cell cultures. In recent years, plant systems have been extensively explored as alternative expression systems that offer safety, cost-effectiveness, scalability (Huang et al., 2009; Thuenemann et al., 2013a; Klimyuk et al., 2014; Mortimer et al., 2015). The potential of plant-based systems has been demonstrated by the approval of the first plant-derived therapeutic for Gaucher’s disease and by the advancement of many plant-made biologics to late-stage clinical development (Gleba et al., 2014). However, the economic feasibility of plant-based systems is strongly yield dependent, and thus, methods of increasing transgene expression are crucial for the success of plants as a recombinant protein production platform.

We and others have previously reported the potential for viral vectors based on bean yellow dwarf virus (BeYDV) to be used for biopharmaceutical production. In this system, the BeYDV replication elements are used to amplify the genes of interest to high copy number in the plant cell nucleus in the form of circular DNA replicons. These replicons utilize the nuclear transcription machinery, leading to the production of large amounts of recombinant protein that is dependent on replication (Huang et al., 2009, 2010; Regnard et al., 2010). Due to the non-competing nature of BeYDV replicons, multiple proteins can be produced in the same cell from the same vector. This contrasts with many RNA virus systems, where the coinfiltration of two different vectors based on the same virus backbone results in one vector being preferentially amplified in a single cell, thus inhibiting the coproduction of multiple proteins in the same cell. This problem has been partially addressed by the identification of TMV and PVX as non-competing viruses for the production of proteins with two heterosubunits (Giritch et al., 2006), however, this system is incapable of producing proteins with more than two heterosubunits. In the BeYDV system, there is presently no known limit to the size or number of proteins that are capable of being efficiently produced (Chen et al., 2011). Additionally, the host range of BeYDV allows the use of these vectors in many dicot plant species, such as tobacco and lettuce (Lai et al., 2012). *Nicotiana* species are the most widely used plant for recombinant protein production due to susceptibility to virus infection, ease of vacuum infiltration, and high biomass (Gleba et al., 2014).

While BeYDV vectors strongly enhance gene expression at the level of transcription, replicon amplification greatly exceeds the enhancement of protein accumulation (Huang et al., 2009, 2010; Regnard et al., 2010). Moreover, for mRNA transcripts to be efficiently utilized, the interplay of multiple post-transcriptional cellular processes is required, many of which are controlled by the regions upstream and downstream of the gene coding sequence. The 5′ untranslated regions (UTR) plays an important role in optimizing transgene production by competing with cellular transcripts for translation initiation factors and ribosomes, increasing mRNA half-life by minimizing mRNA decay or post-transcriptional gene silencing, and avoiding deleterious interactions with regulatory proteins or inhibitory RNA secondary structures (Chiba and Green, 2009; Moore and Proudfoot, 2009; Jackson et al., 2010).

The 5′ UTR from the genomic RNA of tobacco mosaic virus, known as the omega leader, is one of the most well-studied enhancers of translation (Gallie and Walbot, 1992). Several other viral 5′ UTRs have been found to greatly enhance transgene production in many plant systems, including those from alfalfa mosaic virus (AMV; Gehrke et al., 1983), tobacco etch virus (Carrington and Freed, 1990), and pea seadborne mosaic virus (Nicolaisen et al., 1992). Many RNA viruses, such as barley yellow dwarf virus (BYDV), also have 3′ UTRs that contain 3′ cap-independent translation enhancers, which enhance reporter production in tobacco and oat protoplasts (Fan et al., 2012). The combination of the 5′ and 3′ UTRs from cowpea mosaic virus improved protein production in transient expression assays using *Nicotiana benthamiana*, likely due to translational enhancement (Sainsbury et al., 2009). Two native plant 5′ UTRs were identified that improved transgene production at levels comparable to viral 5′ UTRs in transgenic cotton and tobacco (Agarwal et al., 2014). Additionally, a synthetic 5′ UTR was also reported to enhance protein production at a level similar to the TMV 5′ UTR in transgenic tobacco and cotton (Kanoria and Burma, 2012).

Genetic elements downstream from the gene of interest also play a crucial role in optimizing protein production. Proper transcript termination and polyadenylation are necessary for nuclear export, mRNA stability, efficient translation, and prevention of gene silencing (Luo and Chen, 2007; Moore and Proudfoot, 2009). Several terminators have been investigated for their potential to enhance protein production in plants. The 3′ UTR from the potato *pinII* gene was found to enhance hepatitis B virus surface antigen 10–50 fold in transgenic potato compared to the *Agrobacterium*-derived nopaline synthase terminator (Richter et al., 2000). Combining the nopaline synthase terminator with the 35S terminator from cauliflower mosaic virus resulted in a 5–65 fold enhancement of yellow fluorescent protein production compared to the 35S terminator alone (Beyene et al., 2011).

Additionally, chromatin scaffold/matrix attachment regions (MARs) have been explored as genetic elements capable of enhancing transgene production in plant systems. MARs are AT-rich regions thought to be involved in higher-order chromatin structure and that preferentially associate with nuclear matrix, a complex cellular structure with many proposed roles (Liebich et al., 2002; Calikowski et al., 2003; Halweg et al., 2005). Experiments in whole plants and plant cell cultures have shown that the presence of MARs can enhance transcription of flanking genes. The tobacco Rb7 MAR also increased the proportion of plant transformants expressing a transgene (Halweg et al., 2005). Furthermore, MARs have been implicated in the reduction of transgene silencing (Mlynarova et al., 2003). The tobacco TM6 MAR was shown to reduce repressive DNA methylation in flanking promoter regions and enhance recombinant protein production in transgenic tobacco (Ji et al., 2013). An expression vector based on a mild strain of BeYDV that contained the Rb7 MAR has been previously reported, though a comparison to a vector without the MAR was not made (Regnard et al., 2010). The ability for MARs to enhance protein production in transient expression systems has not been thoroughly investigated.

*Agrobacterium*-mediated T-DNA transfer (reviewed in (McCullen and Binns, 2006) is the preferred method of gene delivery in plant transient expression systems (Chen and Lai, 2015). However, *Agrobacterium* is a plant pathogen that has complex effects on infiltrated leaf tissues and often elicits a cell death response (Ditt et al., 2001; Veena et al., 2003). Many studies have found variable effects of different *Agrobacterium* strains, depending on the plant species and system used. One study found that strain GV3101 provided higher transgene expression in *N. benthamiana* and *N. excelsiana* than strains LBA4404, C58C1, at6, at10, at77 and A4 (Shamloul et al., 2014). Additionally, many *Agrobacterium* strains vary greatly in their T-DNA transfer efficiency. Super virulent strains based on strain A281, such as EHA105, were shown to overexpress *virG*, a transcriptional activator which regulates vir gene expression (Jin et al., 1987). Constitutively activated *virG* mutants (Gao et al., 2006) have been used to increase T-DNA transfer efficiency, even when supplied on a separate plasmid (van der Fits et al., 2000). A mutant form of *virD2* was found to enhance gene delivery to tobacco cells (Reavy et al., 2007). These studies suggest there is potential to improve *Agrobacterium* T-DNA transfer and minimize deleterious plant cell interactions.

In the present study, we investigated the potential for diverse genetic elements to enhance protein production using BeYDV vectors. We show that optimizing the 5′ UTR and 3′ transcription terminator region substantially enhances the production of GFP, Norwalk virus capsid protein (NVCP), and the monoclonal antibody rituximab. Further, we demonstrate the potential for a MAR to reduce cell death and enhance protein production in a transient expression system. We also show that the choice of *Agrobacterium* strain can play an important role in plant cell death and recombinant protein yield. Using these optimizations, we have achieved yields of vaccine antigens and monoclonal antibodies equal to or greater than the highest levels ever reported in plant systems.

## Materials and Methods

### Vector Construction

#### Geminiviral Replicon with colE1 Origin of Replication

We constructed a T-DNA backbone vector containing the colE1 origin to enable high-copy replication of plasmids in *Escherichia coli*. The T-DNA vector pGPTV-Kan (Becker et al., 1992) was digested with *Bgl*II and the vector fragment ligated to produce pGPTVKbb containing the pRK2 oriV, *trfA*, and *nptIII* (kanamycin resistance) genes. The colE1 origin from pUC19 was amplified by PCR with primers oriE-Pst-F and oriE-Mlu-R (**Table 1**), digested with *Pst*I-*Mlu*I and ligated with pGPTVKbb digested likewise, to yield pVEKtrf, which was digested with *BspE*I and religated to produce pEKtrf (thus lacking oriV). The oriV segment was amplified by PCR from pGPTV-Kan with primers oriV-Bgl-F and oriV-R1-R, digested with *Bgl*II-*EcoR*I and ligated with pEKtrf digested *Bgl*II-MfeI to give pEKtrfV. A DNA segment containing the *A. tumefaciens* T-DNA left border was inserted by ligation of the 2631 bp *Bgl*II-BspEI fragment from pHB114 (Richter et al., 2000) with pEKtrfV digested likewise, yielding pEKtrfVa. The backbone from pEKtrfVa was incorporated into a geminiviral replicon T-DNA vector by a 3-fragment ligation: pEKtrfVa digested *Pvu*I-*BspE*I, pBYR2p19 (Chen et al., 2011) digested *Pvu*I-*Xba*I (2747 bp), and pBYR2p19-GFP digested *Xba*I-*BspE*I (3677 bp), yielding pBYR2e-GFP. The GFP cds in pBYR2e-GFP was replaced with the pUC19 polylinker (*Xba*I to *Sac*I) by digestion/ligation of both plasmids with *Xba*I-*Sac*I, to make pBYR2eFa.

**Table 1:** Oligonucleotides used in this study.

PsaK1-Xho-F	TACTCGAGCTGAAACAGTCCATTCTGAGGC
PsaK1-Xba-R	GGTCTAGATTTAATTTGCAGCAACTCAACTTTTTTTTCTC
PsaK1T-Xho-F	CCCTCGAGAAAAGCCAATTAAACTAAAAAAAGAAGAG
PsaK2-Xho-F	ATCTCGAGACAAGTATCTTAGTGTATCCAGAATAGCC
PsaK2-Xba-R	GCTCTAGATGTTGCAGAAATTTCAAAGAATTGGAAATGC
PsaK2T-Xho-F	AACTCGAGAAACAAACAAAATCAACAAATATAGAAAATAACG
Ext1	GTGAGCTCGAAGTGACATCACAAAGTTGAAG
Ext2	CAGAATTCGTCATAACTGTAGAAATGATTCC
GFP-f	GTCCAGGAGCGCACCATCTTCT
GFP-r	GATGCCCTTCAGCTCGATGCGGTT
Mar-1	GCGAATTCTCGATTAAAAATCCCAATTATATTTGG
Mar-2	GCGAATTCACTATTTTCAGAAGAAGTTCCC
Mar-Pst1	GCCTGCAGTCGATTAAAAATCCCAATTATATTTGG
Mar-Pst2	GCCTGCAGACTATTTTCAGAAGAAGTTCCC
5D5-F	TCGACATATTGAAGAGACAGAGTGATATATAAAACTGCTAAc
5D5-R	CATGGTTAGCAGTTTTATATATCACTCTGTCTCTTCAATATG
10-F	TCGAAGAATTTTTAGTCAAGAAGTGAc
10-R	CATGGTCACTTCTTGACTAAAAATTCT
12-F	TCGAAGTGGACGTCAATACTTACGCAc
12-R	CATGGTGCGTAAGTATTGACGTCCACT
13-F	TCGAAGATTTAAGTGACGATAAAGTTac
13-R	CATGGtAACTTTATCGTCACTTAAATCT
19-F	TCGAAGTTGTTTTGGATTTAGTCAAGc
19-R	CATGGCTTGACTAAATCCAAAACAACT
20-F	TCGAGGATATGAATGTTGAACAGCTTac
20-R	CATGGtAAGCTGTTCAACATTCATATCC
23-F	TCGAACGAATGCAATCTTGGACGTTAc
23-R	CATGGTAACGTCCAAGATTGCATTCGT
26-F	TCGATGTGAGAATGAATGTTAGCAAAc
26-R	CATGGTTTGCTAACATTCATTCTCACA
43-F	TCGATTATTGCTGAAGTTTTGAGTTAc
43-R	CATGGTAACTCAAAACTTCAGCAATAA
43-Bsa-F	GCGGTCTCCCTAGTTATTGCTGAAGTTTTGAGTTA
48-F	TCGATGAAGAGAAAGTTGAAATTGTAc
48-R	CATGGTACAATTTCAACTTTCTCTTCA
54-F	TCGAAGTGAACTGCAAACGGATTACAc
54-R	CATGGTGTAATCCGTTTGCAGTTCACT
PPT-F	TCGAAAAAGAAGGAAAAAGAAGGGAAGAAAAGGac
PPT-R	CATGGtCCTTTTCTTCCCTTCTTTTTCCTTCTTTT
PSI-F1	TCGAAAAAACAAAAATAAAAAAAACATCGCACAAGAAA
PSI-F2	ATAAAAGATTTGTAGAATCAACTAAGAAACCATG
PSI-R1	CATGGTTTCTTAGTTGATTCTACAAATCTTTTATTTTC
PSI-R2	TTGTGCGATGTTTTTTTTATTTTTGTTTTT
TMV-F	TCGAACAATTACTATTTACAATTACAc
TMV-R	CATGGTGTAATTGTAAATAGTAATTGT
BYDV5-Xho-F	TTCTCGAGTGAAGATTGACCATCTCACAAAAGC
BYDV5-Nco-R	TTCCATGGTGGCGGTGGGGATAGAAGGG
BYDV3-Kpn-F	AAGGTACCAGTGAAGACAACACC
BYDV3-Sac-R	ATGAGCTCGGGTTGCCGAACTGC
PEMV-F1	TCGAGGGTATTTATAGAGATCAGTATGAACTGTGTCGCTAGGATCAAGCGG
PEMV-F2	TGGTTCACACCTGACTTCACCCCTGGCGAGGGCGTGAAGTCTAC
PEMV-R1	CATGGTAGACTTCACGCCCTCGCCAGGGGTGAAGTCAGGTGTGAACCACCGC
PEMV-R2	TTGATCCTAGCGACACAGTTCATACTGATCTCTATAAATACCC
PEMV3-Bsr-F	ATTGTACAAGTAAGGCTTCGCTTCCCGCC
Xho-AMV5-F	TCGAGTTTTTATTTTTAATTTTCTTTCAAATACTTCCAACAT
PSI3′-Xho-F	CGCTCGAGTCGCACAAGAAAATAAAAGATTTG
PSI5′-Xba-R	CCTCTAGATTTTATTTTCTTGTGCGATGTTTT
PSI5′-Nco-R	CCCCATGGTTTTATTTTCTTGTGCGATGTTTT
PSI-Xba-R	GGTCTAGATTTCTTAGTTGATTCTACAAATCTTTTA
5D5-Xba-R	GGTCTAGATTAGCAGTTTTATATATCACTCTGTC
10.10-Xba-R	CGTCTAGATCACTTCTTGACTAAAAATTCTTC
10.43-Xba-R	CGTCTAGATAACTCAAAACTTCAGCAATAATC
10.20-Xba-R	CGTCTAGAtAAGCTGTTCAACATTCATATCC
oriE-Pst-F	CCCTGCAGACCAAGTTTACTCATATATAC
oriE-Mlu-R	CCACGCGTAAAAAGGCCGCGTT
oriV-Bgl-F	GCAGATCTCGACGAGCAAGGCAAGA
oriV-R1-R	GGGGAATTCAATGGCAAGGACTGCC
Ext3i-R	CAATTTGCTTTGCATTCTTGAC
35S-Bsa-F	GCGGTCTCGGCATGGTGGAGCACGA
MAR-Kpn-2	GCGGTACCACTATTTTCAGAAGAAGTTCCC
kpn-f-SIR	GTGGTACCGAGTGTACTTCAAGTCAGTTGG
BAA-Xba-F	CCTCTAGAACAATGGCTAACAAACATCTTTCTTTG
RituxG-Sac-R	CCGAGCTCTTACTTACCAGGTGAAAGAGAC
RituxK-Sac-R	CCGAGCTCTTAGCACTCTCCCCTATTAAAAG

#### 3′ Terminator Constructs

We constructed geminiviral replicons with different 3′ terminator regions downstream of reporter genes. pBYGFP.R (Huang et al., 2009) contains the tobacco etch virus (TEV) 5′UTR and the soybean *vspB* 3′ region flanking the GFP cds. The tobacco (*Nicotiana tabacum*) extensin gene 3′ flanking region, 732 bp including an intron of 226 bp, was amplified by PCR using primers Ext1 and Ext2, which introduced a *Sac*I site at the 5′ end and *EcoR*I site at the 3′ end. After digestion with *Sac*I and *EcoR*I, the extensin 3′ region was substituted for the *vspB* 3′ region in pBYGFP.R to make pBYGFP.REF. Constructs pBYNVCP.R and pBYNVCP.REF were generated by replacing the GFP coding sequence of the pBYGFP.R and pBYGFP.REF with the NVCP cds from psNV210 (Zhang and Mason, 2005) using *Xho*I and *Sac*I sites.

#### 5′UTR Constructs

**FIGURE 1:**
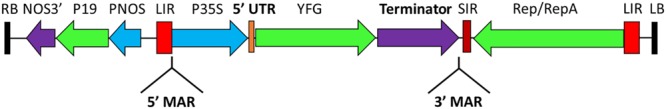
**Vector Map.** Generalized schematic representation of the T-DNA region of the BeYDV vectors used in this study. RB and LB, the right and left borders of the T-DNA region; NOS3′, *Agrobacterium* nopaline synthase 3′ element; P19, tomato bushy stunt virus P19 silencing suppressor; PNOS, *Agrobacterium* nopaline synthase promoter; LIR, long intergenic region of the BeYDV genome; 5′/3′ MAR, tobacco Rb7 matrix attachment region; P35S, 35S promoter from cauliflower mosaic virus; Rep/RepA, C1/C2 ORFs from BeYDV encoding the viral replication proteins. The 5′ UTR, terminator, and 5′/3′ MAR elements are as described in each subsequent section.

We constructed expression vectors having different 5′UTRs linked to reporter genes (**Table 2**). The shuttle cloning vector pBY-GFP212 was constructed by 4-fragment ligation: pBY027 (Mor et al., 2003) digested *Pst*I-*EcoR*I (vector), pBTI210.3 (Judge et al., 2004) digested *Pst*I-*Nco*I (820 bp 35S promoter + TMV 5′UTR), pGFPi210 (Huang et al., 2009) digested *Nco*I-*Sac*I (726 bp GFP cds), and pBYR2p19 digested *Sac*I-*EcoR*I (482 bp tobacco extension 3′ region). Oligonucleotides (**Table 1**) encoding different 5′UTR segments were designed to anneal with 5′ ends compatible with a cut *Xho*I site (5′ protruding TCGA) and 3′ ends compatible with a cut *Nco*I site (5′ protruding CATG). The annealed oligonucleotides were phosphorylated with polynucleotide kinase + ATP, and ligated with pBY-GFP212 digested *Xho*I-*Nco*I to produce the various 5′UTR constructs, pBY-GFP212-XX. The constructs were ligated into pBYR2eFa on *Mfe*I-*Sac*I fragments, to give various P35S-5′UTR-GFP-Ext3′ constructs named pBYR2eXX-GFP (**Figure 1**).

**Table 2:** List of the 5′ UTR DNA sequences used in this study.

Name	Description	Sequence
TMV	Tobacco mosaic virus	GTATTTTTACAACAATTACCAACAACAACAAACAACAAACAACATTACAATTACTATTTACAA
TMV 3′	Tobacco mosaic virus	ACAATTACTATTTACAATTACA
AMV	Alfalfa mosaic virus	TTTTTATTTTTAATTTTCTTTCAAATACTTCCA
TEV	Tobacco etch virus	GAATTAATTCTCAACACAACATATACAAAACAAACGAATCTCAAGCAATCAAGCATTCTACTTC TATTGCAGCAATTTAAATCATTTCTTTTAAAGCAAAAGCAATTTTCTGAAAATTTTCACCATTTACGAACGATAG
5D5	Human-derived	CATATTGAAGAGACAGAGTGATATATAAAACTGCTAA
10	Human-derived	AGAATTTTTAGTCAAGAAGTGA
12	Human-derived	AGTGGACGTCAATACTTACGCA
13	Human-derived	AGATTTAAGTGACGATAAAGTT
19	Human-derived	AGTTGTTTTGGATTTAGTCAAG
48	Human-derived	TGAAGAGAAAGTTGAAATTGTA
54	Human-derived	AGTGAACTGCAAACGGATTACA
20	Human-derived	GGATATGAATGTTGAACAGCTT
23	Human-derived	ACGAATGCAATCTTGGACGTTA
26	Human-derived	TGTGAGAATGAATGTTAGCAAA
43	Human-derived	TTATTGCTGAAGTTTTGAGTTA
PP	Synthetic polypurine	AAAAGAAGGAAAAAGAAGGGAAGAAAAGGG
AtPsaK	*A. thaliana* psaK	AAAAACAAAAATAAAAAAAACATCGCACAAGAAAATAAAAGATTTGTAGAATCAACTAAGAAA
AtPsaK 5′	5′ end of AtPsaK (deletion of nucleotides 1–23)	AAAAACAAAAATAAAAAAAACATCGCACAAGAAAATAAAA
AtPsaK 3′	3′ end of AtPsaK (deletion of nucleotides 42–63)	TCGCACAAGAAAATAAAAGATTTGTAGAATCAACTAAGAAA
NbPsaK1	*N. benthamiana* psaK	CTGAAACAGTCCATTCTGAGGCCACAAACTCCTTGCTTTGGGTAATGGGCCTATGTCACAGA AACTTGTTTGGAACCCCAGTAGATTTATACAAACAATTTTGTCAAAAGCCAATTAAACTAAAA AAAGAAGAGAAAAAAAAGTTGAGTTGCTGCAAATTAAA
NbPsaK1 3′	3′ end of NbPsaK1 (deletion of nucleotides 59–163)	AAAAGCCAATTAAACTAAAAAAAGAAGAGAAAAAAAAGTTGAGTTGCTGCAAATTAAA
NbPsaK2	*N. benthamiana* psaK	ACAAGTATCTTAGTGTATCCAGAATAGCCCCTTCTGTGGCCACAAACTCTTCAAGTGGCCAT GCCACAGAAACTTCTTTCCACCAGAAAAGGGTTTATAACAATTTAAACAAACAAAATCAACA AATATAGAAAATAACGCATTTCCAATTCTTTGAAATTTCTGCAACA
NbPsaK2 3′	3′ end of NbPsaK2 (deletion of nucleotides 75–170	ATAACAATTTAAACAAACAAAATCAACAAATATAGAAAATAACGCATTTCCAATTCTTTGAAATTTCTGCAACA

*Sequences shown here do not include the nucleotides “TCGA” that were added at 5′ to produce a *Xho*I overhang. At 3′, some constructs used “ACC” to accommodate a *Nco*I site, and others used “TCTAGAACA” to accommodate a *Xba*I site.*

Selected constructs were converted to non-replicating vectors by deletion of the BeYDV Rep genes and the downstream LIR, accomplished by digesting with *Bam*HI-*Avr*II, filling the recessed 3′ ends with Klenow fragment DNA polymerase, and ligating the vector fragment, to give plasmids named pBYL2eXX-GFP. A non-replicating construct with TMV 5′UTR was constructed by ligation of pBYL2e20-GFP digested *Mfe*I-*Sac*I (vector) and pBYR2e-GFP digested *Mfe*I-*Sac*I (1150 bp) to yield pBYL2eFc-GFP. Truncations of the *A. thaliana* psaK (PSI) 5′UTR in non-replicating vectors were made. PCR amplification of pBYL2ePSIa-GFP with primers PSI3′-Xho-F and Ext3i-R, digestion of the product with *Xho*I-*Sac*I, and insertion into pBYL2eFc-GFP digested *Xho*I-*Sac*I yielded pBYL2ePSI3′-GFP, containing the 3′ 41 nt of the 5′UTR. A similar deletion of the 3′ end was produced by PCR amplification of pBYL2ePSIa-GFP with primers 35S-Bsa-F and PSI5′-Xba-R, digestion of the product with *Mfe*I-*Xba*I, and insertion into pBYL2eFc-GFP digested *Mfe*I-*Xba*I to make pBYL2ePSI5′-GFP. Replicating vectors containing the 3′ 41 nt of the AtPsaK 5′ UTR were generated by digesting pBYL2ePSI3′-GFP with *Xba*I-*Fse*I (vector fragment) and inserting the *Xba*I-*Fse*I fragment from either pBYR2e-GFP or pBYR2eFa-sNV to generate pBYR2eP3-GFP and pBYR2eP3-sNV respectively.

Homologs of *A. thaliana* psaK were identified using the Sol Genomics *N. benthamiana* draft genome (https://solgenomics.net/organism/Nicotiana_benthamiana/genome). pBYR2e-GFP was digested *Xho*I-*Sac*I and the GFP fragment was inserted into psNV120e (a non-replicating T-DNA vector; details available upon request) digested *Xho*I-*Sac*I yielding pGFPe-TMV. The upstream region from the first psaK homolog, referred to as NbPsaK1, was PCR amplified from *N. benthamiana* genomic DNA using primers PsaK1-Xho-F and PsaK1-Xba-R, and the second homolog, referred to as NbPsaK2, was amplified similarly using primers PsaK2-Xho-F and PsaK2-Xba-R. The PCR fragments were digested with *Xho*I-*Xba*I and ligated into pGFPe-TMV digested *Xho*I-*Xba*I yielding pGFPe-NbPsaK1 and pGFPe-NbPsaK2. A truncation of the NbPsaK1 5′ UTR was generated by PCR amplifying pGFPe-NbPsaK1 with primers PsaK1T-Xho-F and Ext3i-R, digestion of the product with *Xho*I-*Sac*I, and insertion into pGFPe-TMV digested *Xho*I-*Sac*I to yield pGFPe-NbPsaK1T. pGFPe-NbPsaK2T was created similarly using PCR primers PsaK2T-Xho-F and Ext3i-R, followed by *Xho*I-*Sac*I digestion and insertion into pGFPe-TMV digested *Xho*I-*Sac*I.

Selected 5′UTRs were modified to contain *Xba*I sites at the 3′ end for fusion with the NVCP cds. The shuttle vectors (pBY-GFP212-XX) were amplified by PCR with reverse primers containing an *Xba*I site and M13-R, and the resulting products digested *Pst*I-*Xba*I and ligated with pBYR2eFa-sNV digested likewise, thus yielding the various vectors names *pBYR2eXX-sNV*.

The rituximab heavy chain was obtained by PCR amplifying pMAP-RitX-G1-B (a kind gift from Mapp Biopharmaceuticals, San Diego, CA, USA) with primers BAA-Xba-F and RituxG-Sac-R. The resulting PCR fragment was digested with *Xba*I-*Sac*I and ligated into a derivative of pBY027 (Mor et al., 2003), digested likewise, yielding pBYR0-LRtxGT. The rituximab light chain was similarly cloned by amplifying pMap-RitX-K-b (Mapp Biopharmaceuticals) with BAA-Xba-F and RituxK-Sac-R, digested *Xba*I-*Sac*I, and ligated into a derivative of pBY027 digested likewise to yield pBYR0-LRtxKF. To generate T-DNA vectors, the rituximab heavy chain was obtained by *Xho*I-*Sac*I digestion of pBYR0-LRtxGT and inserted into pBYR2e-GFP (vector) digested *Xho*I-*Sac*I to yield pBYR2e-MRtxG. The rituximab light chain was obtained by *Xho*I-*Sac*I digestion of pBYR0-LRtxKF and inserted into pBYR2e-GFP digested *Xho*I-*Sac*I to yield pBYR2e-MRtxK. The AtPsaK 5′ UTR fused to the rituximab heavy and light chains was obtained by digesting pBYR0-LRtxGT XbaI-SacI (heavy chain) or pBYR0-LRtxKF *Xba*I-*Sac*I (light chain) and ligating into pBYR2ePSI-GFP (vector) digested *Xba*I-*Sac*I to yield pBYR2ePSI-MRtxG and pBYR2ePSI-MRtxK respectively.

#### MAR Constructs

The tobacco Rb7 MAR was PCR amplified from genomic DNA using primers Mar-1 and Mar-2 designed to create *EcoR*I sites on either end. The amplified fragment was digested with *EcoR*I and ligated into pBY027 digested likewise to yield pBY027-MAR. pBY027-MAR was PCR amplified with primers Mar-1 and Mar-Kpn-2 to create a KpnI site on the 3′ end. To generate a *Kpn*I in the BeYDV vector, primers LIR-R and Kpn-F-SIR were used to amplify the LIR-C1/C2-SIR segment of pBYGFP.REF. A 3-fragment ligation consisting of pBYGFP.REF (vector) digested *Hind*III-*EcoR*I, the *Hind*III-*Kpn*I digested segment of the LIR-C1/C2-SIR PCR product, and the *Kpn*I-*EcoR*I digested MAR fragment was used to make pBYR-GEM. To create the 5′ MAR, pBY027-MAR was PCR amplified with primers Mar-Pst1 and Mar-Pst2. The product was digested with *Pst*I and ligated into pBYR-GEM digested with *Sbf*I to make pBYR-MGEM. The *Sac*I-*Fse*I fragment containing the 3′ MAR from pBYR-MGEM was ligated into vectors containing the rituximab heavy chain (pBYR2e-MrtxG) or light chain (pBYR2e-MRtxK) to yield pBYR2e-MRtxGM and pBYR2e-MRtxKM respectively. The 5′ + 3′ MAR rituximab construct was created by digesting pBYR-MGEM with *Xho*I-*Asc*I to obtain the 5′ MAR fragment, and ligating it into pBYR2e-MRtxG or pBYR2e-MRtxK, yielding pBYR2e-MMGM and pBYR2e-MMKM respectively.

### Agroinfiltration of *Nicotiana benthamiana* Leaves

Binary vectors were separately introduced into *Agrobacterium tumefaciens* LBA4404, LBA4301, GV3101, or EHA105 by electroporation. The resulting strains were verified by restriction digestion or PCR of plasmid DNA, grown overnight at 30°C, and used to infiltrate leaves of 5- to 6-week-old *N. benthamiana* maintained at 23–25°C. Briefly, the bacteria were pelleted by centrifugation for 5 min at 5,000 *g* and then resuspended in infiltration buffer [10 mM 2-(*N*-morpholino)ethanesulfonic acid (MES), pH 5.5 and 10 mM MgSO4] to OD600 = 0.2. The resulting bacterial suspensions were injected by using a syringe without needle into leaves through a small puncture (Huang and Mason, 2004). For antibody coinfiltrations, *Agrobacterium* suspensions were mixed such that the final concentration of each corresponded to OD600 = 0.2. Plant tissue was harvested at 4 DPI unless otherwise noted.

### Protein Extraction

Total protein extract was obtained by homogenizing agroinfiltrated leaf samples with 1:5 (*w:v*) ice cold extraction buffer (25 mM sodium phosphate, pH 7.4, 100 mM NaCl, 1 mM EDTA, 0.2% Triton X-100, 10 mg/mL sodium ascorbate, 10 mg/mL leupeptin, 0.3 mg/mL phenylmethylsulfonyl fluoride) using a Bullet Blender machine (Next Advance, Averill Park, NY, USA) following the manufacturer’s instruction. To enhance solubility, homogenized tissue was rotated at room temperature for 30 min. The crude plant extract was clarified by centrifugation at 10,000 *g* for 10 min at 4°C. Protein concentration of clarified leaf extracts was measured using a Bradford protein assay kit (Bio-Rad) with bovine serum albumin as standard.

### SDS-PAGE

For SDS-PAGE, clarified plant protein extract was mixed with sample buffer (50 mM Tris-HCl, pH 6.8, 2% SDS, 10% glycerol, 200 mM dithiothreitol, 0.02 % bromophenol blue), boiled for 10 min, and separated on 4–15% polyacrylamide gels (Bio-Rad). For GFP fluorescence, PAGE gels were visualized under UV illumination (365 nm). PAGE gels were stained with PageBlue protein staining solution (Thermo Fisher) or Coomassie stain (Bio-Rad) following the manufacturer’s instructions. Following protein staining, the 26 kDa band corresponding to GFP or the 58 kDa band corresponding to NVCP were analyzed using ImageJ software to quantify the band intensity.

### ELISA

NVCP concentration was analyzed by sandwich ELISA as described (Mason et al., 1996). Briefly, a rabbit polyclonal anti-NVCP antibody was bound to 96-well high-binding polystyrene plates (Corning), and the plates were blocked with 5% non-fat dry milk in PBS. After washing the wells with PBST (PBS with 0.05% Tween 20), the plant extracts were added and incubated. The bound NVCP were detected by incubation with guinea pig polyclonal anti-NVCP antibody followed by goat anti-guinea pig IgG-horseradish peroxidase conjugate. The plate was developed with TMB substrate (Pierce) and the absorbance was read at 450 nm. Plant-produced NVCP was used as the reference standard (Kentucky BioProcessing).

For rituximab quantification, plant protein extracts were analyzed by ELISA designed to detect the assembled form of mAb (with both light and heavy chains) as described previously (Giritch et al., 2006). Briefly, plates were coated with a goat anti-human IgG specific to gamma heavy chain (Southern Biotech, Birmingham, AL, USA). After incubation with plant protein extract, the plate was blocked with 5% non-fat dry milk in PBS, then incubated with a HRP-conjugated anti-human-kappa chain antibody (Southern Biotech) as the detection antibody. Human IgG was used as a reference standard (Southern Biotech).

### GFP Fluorescence

Leaves producing GFP were photographed under UV illumination generated by a B-100AP lamp (UVP, Upland, CA, USA). The GFP fluorescence intensity was examined on a microplate reader (Molecular Device Co, Spectra Max M2). GFP samples were prepared by serial twofold dilution with phosphate buffered saline (PBS, 137 mM NaCl, 2.6 mM KCl, 10 mM Na_2_HPO_4_, and 1.8 mM KH_2_PO_4_, pH 7.4) and 50 μl of each sample was added to black-wall 96-well plates (Corning), in duplicate. The excitation and emission wavelength were 485 and 538 nm, respectively. All measurements were performed at room temperature and the reading of an extract from an uninfiltrated plant leaf was subtracted before graphing. *E. coli* expressed GFP was used to generate the standard curve. GFP gene was cloned into the pET28 expression vector (Invitrogen) and IPTG-induced GFP was purified using TALON His-Tag purification resin (Clontech).

### cDNA Synthesis and Quantitative RT-PCR

Total RNA was prepared using Plant RNA Reagent (Invitrogen) according to the manufacturer’s protocol and residual DNA was removed using the DNAfree system (Ambion). Aliquots of 1 μg of total RNA were subjected to first-strand cDNA synthesis with oligo dT20 primer using a Superscript III First-Strand Synthesis System (Invitrogen) according to the manufacturer’s instructions: a 10 μl reaction was prepared using 1 μg total RNA, 100U Superscript III Reverse Transcriptase (Invitrogen) and 50 pmol oligo dT20 primers. The reaction as carried out for 50 min at 50°C, and then 5 min at 85°C to deactivate the enzyme. The cDNA was stored at -20°C. Quantitative RT-PCR was performed on an IQ5 Real-Time PCR Detection System (Bio-Rad). For GFP transcripts, gene specific primers (GFP-f and GFP-r) and custom made Taqman probe (GFP-pro, Integrated DNA Technologies) were used. As an internal control, *N. benthamiana* translation elongation factor 1 alpha (EF1a, accession number AY206004) was used (primers EF1f, EF1r and EF1p). Each sample was measured in triplicate for GFP transcripts and an internal reference gene and compared to a standard curve using purified plasmid DNA. Reactions contained in 25 μl: 2 μl of 1:10 diluted cDNA, 2.5 μl of 10X Ex Taq buffer (20 mM MgCl_2_), 1 μl of 10 μM each gene-specific primers, 0.5 μl of 10 μM gene specific probe labeled with FAM (Integrated DNA Technologies), 0.2 μl of Ex Taq polymerase (5 U/μl) and 17.8 μl of distilled water. PCR conditions were: 50°C for 2 min, 95°C for 10 min, followed by 40 cycles at 95°C for 15 s and 55°C for 30 s. Relative quantification of target gene transcript was estimated using standard curve of Ct values generated using 10-fold serial dilution of plasmid DNA.

### Statistical Analysis

For each experiment, plants of the same age were used to minimize developmental differences. Additionally, experiments were designed to compare each construct directly on the same leaf to minimize leaf-to-leaf variation. Comparisons between leaves were made using leaves of similar developmental stage. Data are presented as mean ± SE. Three or more independent infiltrations were made for each experiment and compared using Student’s *t*-test (two-tailed). *P* < 0.05 was represented with two stars (^∗∗^) and *P* < 0.01 was represented with three stars (^∗∗∗^).

## Results

### Tobacco Extensin Terminator Enhances Transgene mRNA Accumulation and Protein Production

We previously reported BeYDV vectors that contained the soybean *vspB* terminator following the gene of interest (Huang et al., 2009). We tested several 3′ elements and identified the tobacco extensin terminator as an efficient transcription terminator and a potent enhancer of transgene expression, as compared to the nopaline synthase, 35S, and other commonly used terminators (data to be presented elsewhere). Extensin is a hydroxyproline-rich glycoprotein that constitutes the major protein component of cell walls.

**FIGURE 2:**
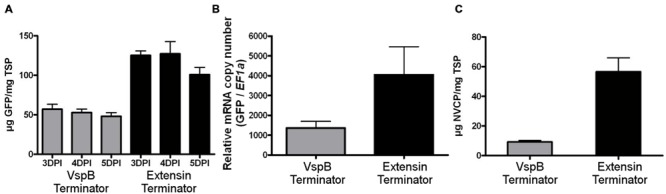
**Extensin terminator increases transgene production and mRNA accumulation. (A)** Fluorimetric analysis of GFP production. *N. benthamiana* leaves were agroinfiltrated with BeYDV vectors containing either the *vspB* terminator (gray bars, pBYGFP.R) or tobacco extensin terminator (black bars, pBYGFP.EFR). Leaves from agroinfiltrated tissue were harvested at 3–5 DPI and analyzed for GFP production by spectrofluorimetry using excitation and emission wavelengths of 485 and 538 nm. GFP production was normalized by total soluble protein. Columns represent means ± SD from six independently infiltrated samples. **(B)** Total RNA from agroinfiltrated *N. benthamiana* leaves were analyzed for GFP mRNA accumulation by quantitative RT-PCR at 3 DPI. Translation elongation factor 1α was used as an internal loading control. Columns represent means ± SD from four independent infiltrated samples. **(C)** NVCP production. Protein extracts from *N. benthamiana* leaves agroinfiltrated with either the *vspB* terminator (gray bars, pBYNVCP.R) or the tobacco extensin terminator (black bars, pBYNVCP.REF) harvested at 3 DPI were analyzed for NVCP production by ELISA and normalized by total soluble protein. Columns represent means ± SD for nine independently infiltrated samples.

To test the potential of the tobacco extensin terminator to enhance protein production in the BeYDV system, *N. benthamiana* leaves were agroinfiltrated with replicating GFP vectors containing either the soybean *vspB* terminator or the tobacco extensin terminator (**Figure 1**). Protein extracts from agroinfiltrated leaf samples were analyzed from 3 to 5 DPI by spectrofluorimetry. Vectors containing the tobacco extensin terminator provided an approximately 2.5-fold increase in GFP production compared to *vspB* (**Figure 2A**). Quantitative real-time RT-PCR showed that the increase in GFP was associated with a similar 2.5-fold increase in GFP transcripts (**Figure 2B**).

Next, we wanted to determine whether the enhancing effect of the tobacco extensin terminator was gene-specific. Vectors producing NVCP were agroinfiltrated and analyzed by ELISA. NVCP concentration was normalized by total soluble protein (TSP) to eliminate differences in the extraction efficiency or leaf water weight. An approximately sixfold increase in NVCP production was observed with the tobacco extensin terminator compared to *vspB* (**Figure 2C**). We have also shown that the tobacco extensin terminator improves the production of monoclonal antibodies by a similar level (data not shown). These data indicate that the tobacco extensin terminator has strong potential to enhance transgene expression, likely by stabilization of the mRNA, and its enhancing effect is not gene-specific. The tobacco extensin terminator was used for all further studies.

### Diverse 5′ UTRs Greatly Impact Transgene Production

Previously, we reported BeYDV vectors that contained the 5′ UTR from either tobacco etch virus (TEV) or tobacco mosaic virus (TMV). In order to systematically evaluate the role of the 5′ UTR on transgene production, we created a series of BeYDV transient expression vectors containing diverse 5′ UTRs from viral, plant, and human sources upstream from the green fluorescent protein (GFP) gene (**Figure 1**; **Table 2**). As the nucleotides directly surrounding the start codon are known to play a role in translation initiation, we standardized all vectors to contain the nucleotides ACC (to accommodate the *Nco*I site) or ACA preceding the ATG, which has been reported to be optimal for dicot plants (Sugio et al., 2010). We found no difference in the performance of vectors with ACC or ACA. These vectors were delivered to *N. benthamiana* leaves by agroinfiltration and monitored for green fluorescence. To minimize leaf-to-leaf variation, each leaf was infiltrated with a vector containing the TMV 5′ UTR as an internal control alongside vectors containing the 5′ UTRs to be tested.

**FIGURE 3:**
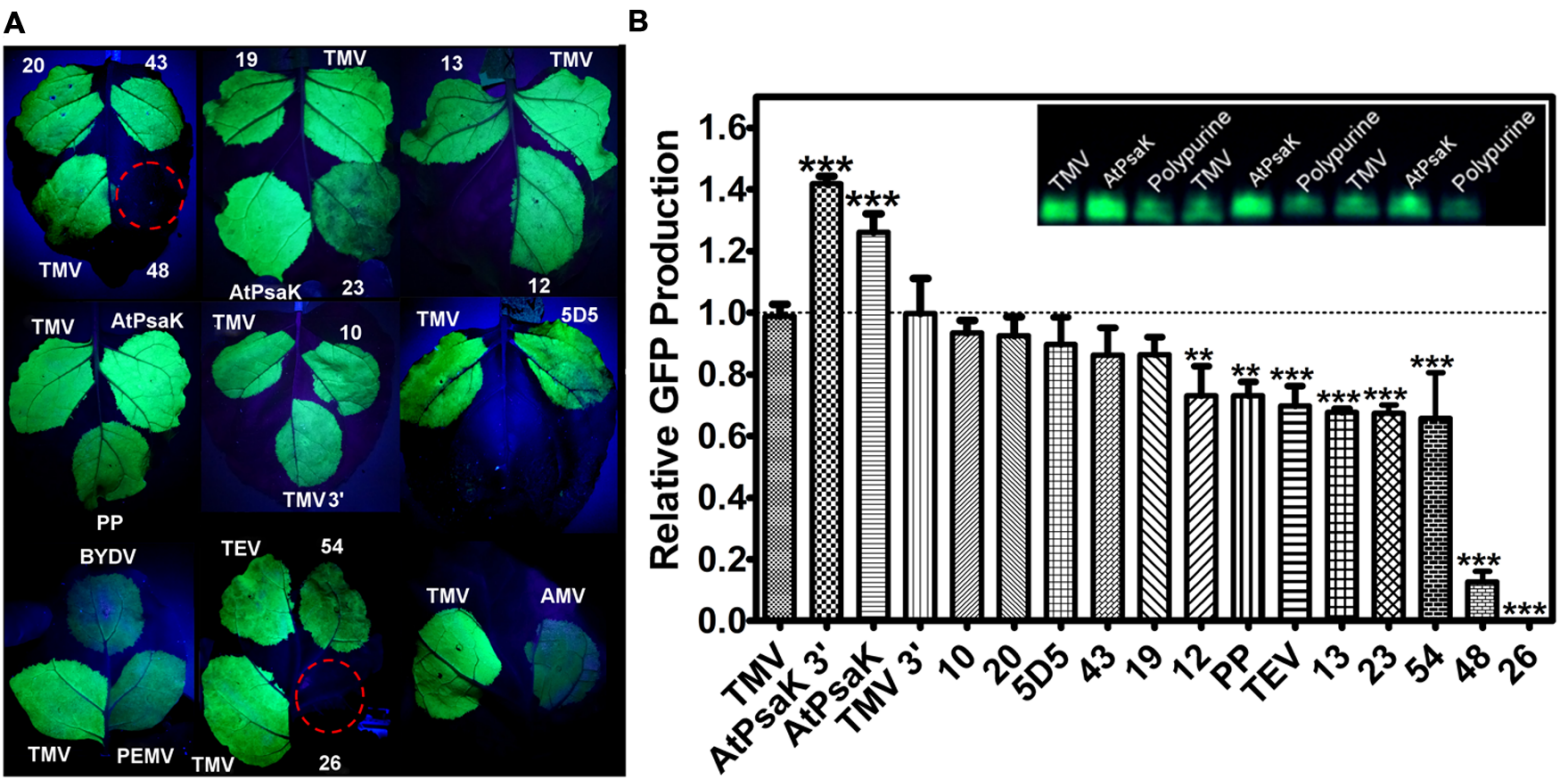
**Evaluation of diverse 5′ UTRs on GFP production.** Leaves of *N. benthamiana* were agroinfiltrated with BeYDV containing different 5′ UTRs upstream from the GFP gene vectors (vector pBYR2eXX-GFP, where XX denotes the individual 5′ UTRs). **(A)** Leaves were photographed at 4 DPI under UV illumination (365 nm). Images are representative of 3–4 independently infiltrated leaves. **(B)** Agroinfiltrated leaves were harvested between 4 and 5 DPI and extracts were analyzed by SDS-PAGE followed by observation under UV illumination (365 nm) and Coomassie staining. GFP band intensity was quantified using ImageJ software, using native plant proteins as a loading control. Columns represent means ± standard error of three or more independently infiltrated leaves. All leaves were infiltrated with the TMV 5′ UTR vector in addition to the other vectors as an internal control for leaf and plant variability. Two stars (^∗∗^) indicate *p* < 0.05 and three stars (^∗∗∗^) indicate *p* < 0.01 as compared to TMV by Student’s *t*-test. 5′ UTR key (position -1 taken as first nucleotide upstream from ATG): TMV, tobacco mosaic virus full length 5’ UTR; AtPsaK 3′, nucleotides -1 to -41 of AtPsaK gene; AtPsaK, nucleotides -1 to -63 of AtPsaK gene; TMV 3′, nucleotides -1 to -21 of TMV; TEV, tobacco etch virus full length 5′ UTR; PP, synthetic polypurine sequence; AMV, full length alfalfa mosaic virus 5′ UTR; BYDV, full length barley yellow dwarf virus 5′ and 3′ UTRs; PEMV, full length pea enation mosaic virus RNA 2 5′ and 3′ UTRs; 10; 20; 5D5; 43; 19; 12; 13; 23; 54; 48; 26, human-derived 5′ UTR sequences.

First, we compared a set of 11 human-derived sequences found to provide cap-independent translational enhancement (Wellensiek et al., 2013). There is evidence that some 5′ UTR elements function cross-kingdom, especially A-rich polypurine sequences (Dorokhov et al., 2002; Terenin et al., 2005). We found that many of the human 5′ UTRs, as well as a polypurine sequence, produced bright green fluorescence under UV illumination (**Figure 3A**). To further analyze GFP production, protein extracts from agroinfiltrated leaves were separated by SDS-PAGE followed by Coomassie staining or visualization under UV light, and the GFP band intensity was quantified by densitometry. Gel quantification showed many of the human-derived 5′ UTRs, as well as the polypurine 5′ UTR, produced GFP at a level comparable to the commonly used plant viral 5′ UTRs from TEV and TMV (**Figure 3B**). These data indicate that 5′ UTRs from sources outside of the plant kingdom can support high levels of translation in *N. benthamiana*.

Next, we tested the 5′ UTRs from RNA plant viruses. Among the 5′ UTRs tested, the TMV 5′ UTR appeared to provide the brightest fluorescence, followed by TEV (**Figure 3A**). Using gel quantification, the TMV 5′ UTR provided a >40% increase in GFP yield over the TEV 5′ UTR (**Figure 3B**). A truncation containing only nucleotides 1–22 of the TMV 5′ UTR performed as well as the full length sequence, indicating that the poly(CAA) region of the TMV 5′ UTR is not necessary for high levels of translation in *N. benthamiana*, at least in a replicating system (**Figure 3B**). Constructs containing the 5′ UTR from AMV, as well as constructs containing the 5′ and 3′ UTRs from BYDV or pea enation mosaic virus (PEMV; Fan et al., 2012), showed poor GFP production and were not studied further (**Figure 3A**).

**FIGURE 4:**
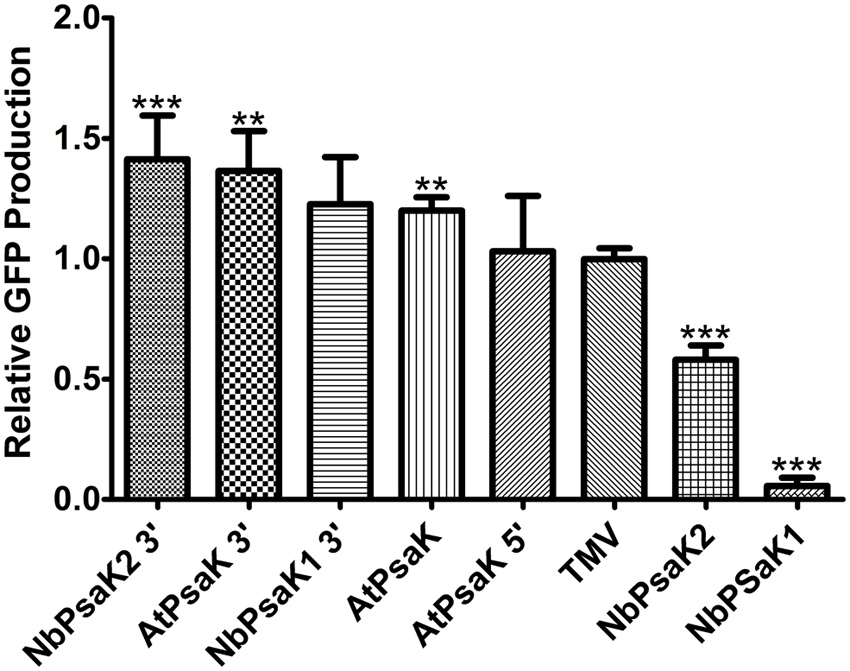
**Truncated psaK 5′ UTRs provide high levels of transgene production.** Leaves of *N. benthamiana* were agroinfiltrated with non-replicating vectors containing different psaK 5′ UTRs, or the TMV 5′ UTR, upstream from the GFP gene (pGFPe-XX, where XX denotes the 5′ UTR). Agroinfiltrated leaf tissue was harvested at 5 DPI and leaf extracts were analyzed by SDS-PAGE followed by coomassie staining. GFP band intensity was quantified using ImageJ software, using native plant protein bands as a loading control. Columns represent means ± standard error from four independently infiltrated samples. Two stars (^∗∗^) indicate *p* < 0.05 and three stars (^∗∗∗^) indicate *p* < 0.01 as compared to TMV by Student’s *t*-test.

We also wished to test the activity of plant-derived 5′ UTRs in the BeYDV system. It has been reported that a 5′ UTR derived from 63 nucleotides upstream from the start codon of the *A. thaliana* photosystem K subunit (AtPsaK) enhanced transgene expression in transgenic tobacco leaves (Agarwal et al., 2014). Using our system, we found that the 63nt AtPsaK 5′ UTR produced intense green fluorescence, and gel quantification data indicate that GFP production was increased by >20% compared to the TMV 5′ UTR (**Figures 3A,B**). The AtPsaK 5′ UTR (accession NM_102775) appears to be a truncation of the full-length 129 nt 5′ UTR. To further delineate the active region of the AtPsaK 5′ UTR, we created deletions at its 5′ and 3′ ends and tested their potential to enhance GFP production in non-replicating transient expression vectors. Using gel quantification, we found that a truncation removing nucleotides -1 to -23 upstream from the start codon resulted in a ∼14% decrease in GFP production, while a truncation removing nucleotides -42 to -63 resulted in a ∼13% increase in GFP production (**Figure 4**). A similar ∼12% enhancement was observed in replicating GFP vectors (**Figure 3B**).

To determine whether the 5′ UTRs from related psaK genes have potential for high levels of transgene production, two psaK homologs were identified from *N. benthamiana* (referred to as NbPsaK1 and NbPsaK2) and their 5′ upstream regions were cloned into non-replicating GFP expression vectors. These vectors were agroinfiltrated alongside the TMV and AtPsaK 5′ UTRs for comparison. By gel quantification, the 163 nt upstream region from NbPsaK1 was found to have very minimal activity, whereas the 170 nt upstream region from NbPsaK2 produced GFP at ∼50% of the level of the TMV 5′ UTR (**Figure 4**). Inspection of the nucleotide sequence revealed the presence of upstream ATGs in both NbPsaK1 and NbPsaK2. As the 3′ end was the most active region of the AtPsaK 5′ UTR, similar truncations were made for the NbPsaK upstream regions (referred to as NbPsaK1 3′ and NbPsaK2 3′). These new constructs were agroinfiltrated alongside the full-length version and tested by gel quantification. The NbPsaK1 truncation enhanced GFP production by >20-fold compared to the original vector (**Figure 4**). The NbPsaK2 truncation enhanced GFP production by 2.4-fold compared to the original vector, corresponding to a >40% improvement compared to the TMV 5′ UTR (**Figure 4**). These results indicate that the regions 40–60 nt upstream from the *A. thaliana* and *N. benthamiana* psaK genes are highly active in *N. benthamiana* leaves, and are capable of enhancing protein production at a level greater than the widely used TMV 5′ UTR.

**FIGURE 5:**
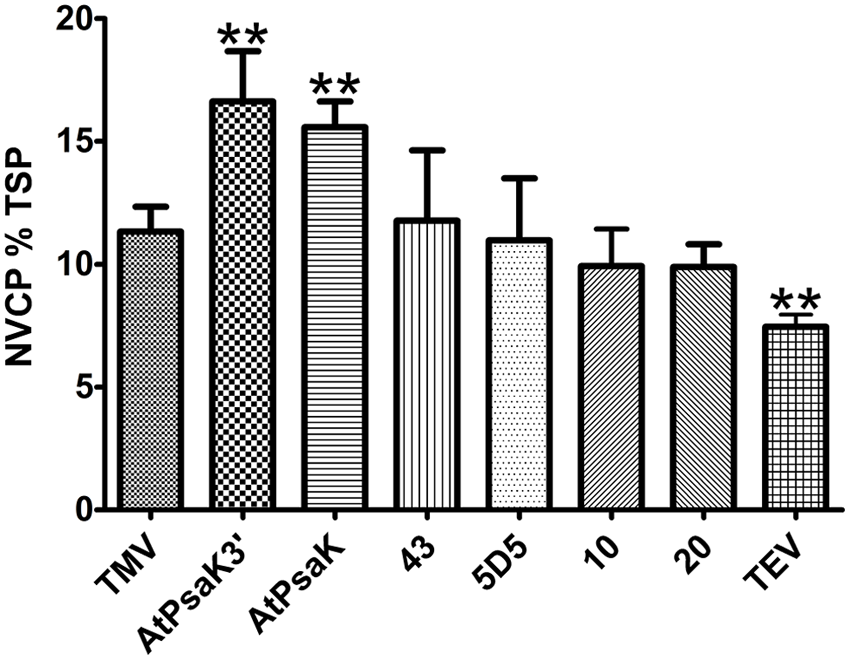
**Evaluation of 5′ UTRs on NVCP production.** Agroinfiltrated leaves of *N. benthamiana* were harvested between 4 and 5 DPI and protein extracts were analyzed for NVCP production (vector pBYR2eXX-sNV) by ELISA. NVCP concentration in leaf extracts was normalized by total soluble protein. Columns represent results from four independently infiltrated leaf samples ± standard error. Two stars (^∗∗^) indicate *p* < 0.05 as compared to TMV by Student’s *t*-test.

To further assess the potential of the 5′ UTR to improve transgene production, several promising 5′ UTRs were tested in BeYDV vectors producing NVCP. Protein extracts from agroinfiltrated leaf samples were normalized for TSP and analyzed by NVCP ELISA. In general agreement with the results found for GFP, several of the human 5′ UTRs performed as well as the TMV 5′ UTR, and the TMV 5′ UTR resulted in a ∼30% increase in NVCP production compared to the TEV 5′ UTR (**Figure 5**). Additionally, vectors containing the AtPsaK 5′ UTR produced NVCP at 15.9 ± 1.5% TSP compared to 11.3 ± 1.0% TSP for the TMV 5′ UTR (**Figure 5**). Further, the truncated form of the AtPsaK 5′ UTR (AtPsaK 3′) produced as much or more NVCP as the unmodified 5′ UTR. These data further demonstrate the capacity of the 5′ UTR to enhance recombinant protein production, and show that the enhancing activity of the unmodified or truncated AtPsaK 5′ UTR is not gene-specific.

### Matrix Attachment Regions Enhance Transgene Production and Reduce Plant Cell Death

The presence of MARs has been reported to enhance transgene production using transgenic systems (Halweg et al., 2005; Xue et al., 2005; Ji et al., 2013). Many of the postulated mechanisms by which MARs enhance transgene production, such as by preventing repressive chromatin modifications or by the interaction of chromatin with the nuclear matrix, require the gene of interest to be organized into chromatin. Thus it is unclear whether MARs would function in transient expression systems that do not involve stable chromosomal integration. However, replicated geminivirus DNA has been shown to associate with cellular histones, forming viral minichromosomes (Pilartz and Jeske, 1992, 2003). Therefore, we investigated the potential for MARs to improve BeYDV vectors.

**FIGURE 6:**
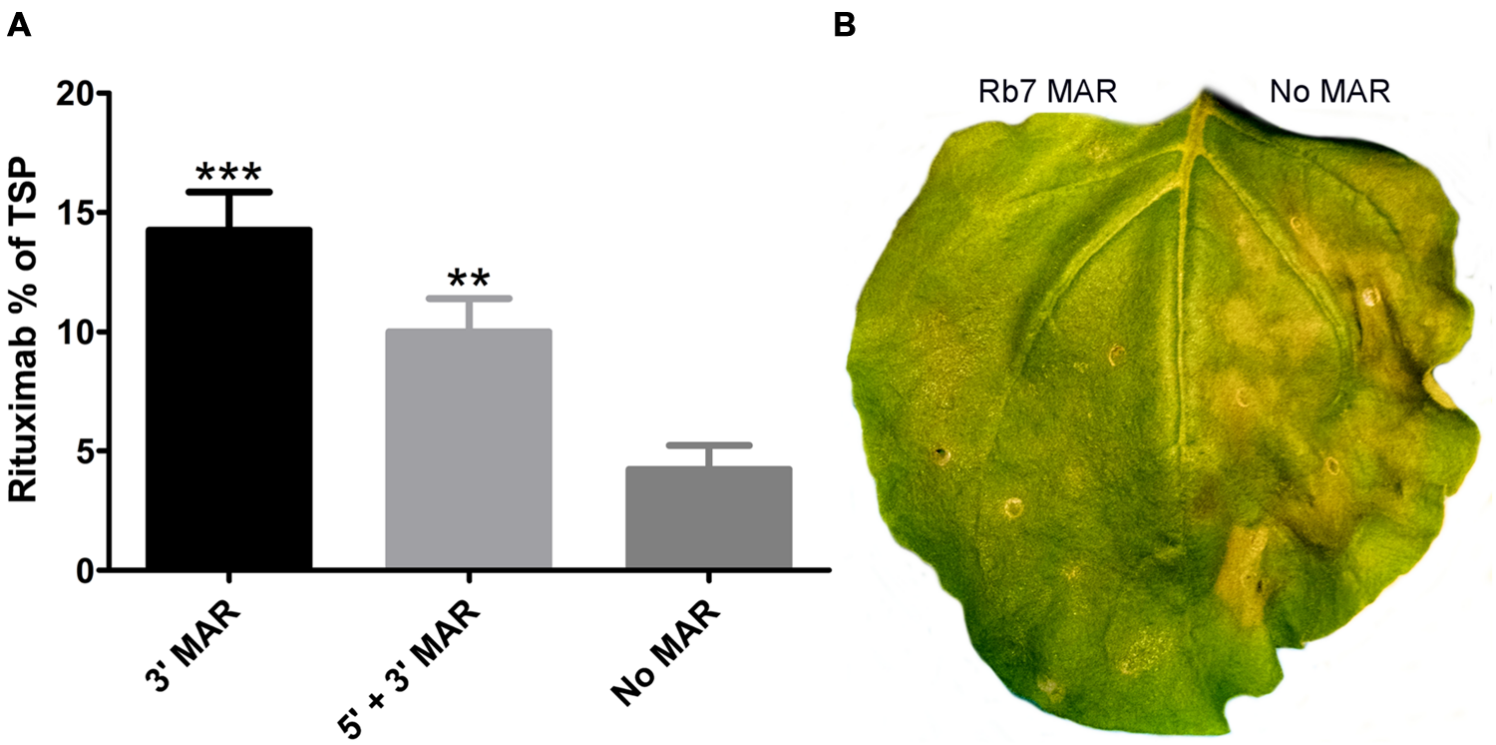
**Rb7 MAR enhances transgene production and reduces cell death. (A)** Protein extracts from leaves of *N. benthamiana* were infiltrated with BeYDV vectors containing the rituximab heavy chain and light chain were analyzed by ELISA. Constructs contained either the Rb7 MAR both upstream from the promoter and downstream from the terminator (5′ + 3′ MAR, vectors pBYR2e-MMGM and pBYR2e-MMKM); the downstream Rb7 MAR only (3′ MAR, vectors pBYR2e-MrtxGM and pBYR2e-MrtxKM); or no modifications (No MAR, vectors pBYR2e-MRtxG and pBYR2e-MRtxK). Rituximab concentrations were normalized by total soluble protein. Columns represent results from four independently infiltrated leaf samples ± standard error. Two stars (^∗∗^) indicate *p* < 0.05 and three stars (^∗∗∗^) indicate *p* < 0.01 as compared to construct No MAR by Student’s t-test. **(B)** *N. benthamiana* leaves were agroinfiltrated with BeYDV rituximab vectors either containing 5′ + 3′ MAR (left half of leaf) or no MAR (right half of leaf). A representative leaf was photographed under visible light at 4 DPI.

The tobacco Rb7 MAR was inserted into BeYDV vectors (**Figure 1**) either with two copies flanking the expression cassette (5′ + 3′ MAR), or one copy in the 3′ position (3′ MAR). Placing the MAR only in the 5′ position was not found to be as effective as the other two configurations in preliminary studies and was not pursued further (data not shown). Leaves of *N. benthamiana* were co-infiltrated with BeYDV vectors containing the rituximab heavy and light chains both either with or without the Rb7 MAR at either the 3′ or 5′ + 3′ positions. Protein extracts from infiltrated leaf spots were normalized for TSP and assayed for rituximab production by IgG ELISA. Remarkably, it was found that while both MAR-containing vectors enhanced IgG production, the vector containing only the 3′ MAR resulted in a 3.4-fold increase in IgG production, representing 14.3 ± 1.6% TSP for the 3′ MAR vector compared to 4.2 ± 1% TSP for the control with no MAR elements (**Figure 6A**). Inspection of the infiltrated leaves revealed a substantial reduction in leaf tissue necrosis with the MAR-containing vectors (**Figure 6B**). These data indicate that MARs have potential to enhance protein production using geminiviral transient expression vectors, and this enhancement is correlated with a reduction in plant cell death.

### Effects of *Agrobacterium* Strain on Transgene Production and Cell Death

**FIGURE 7:**
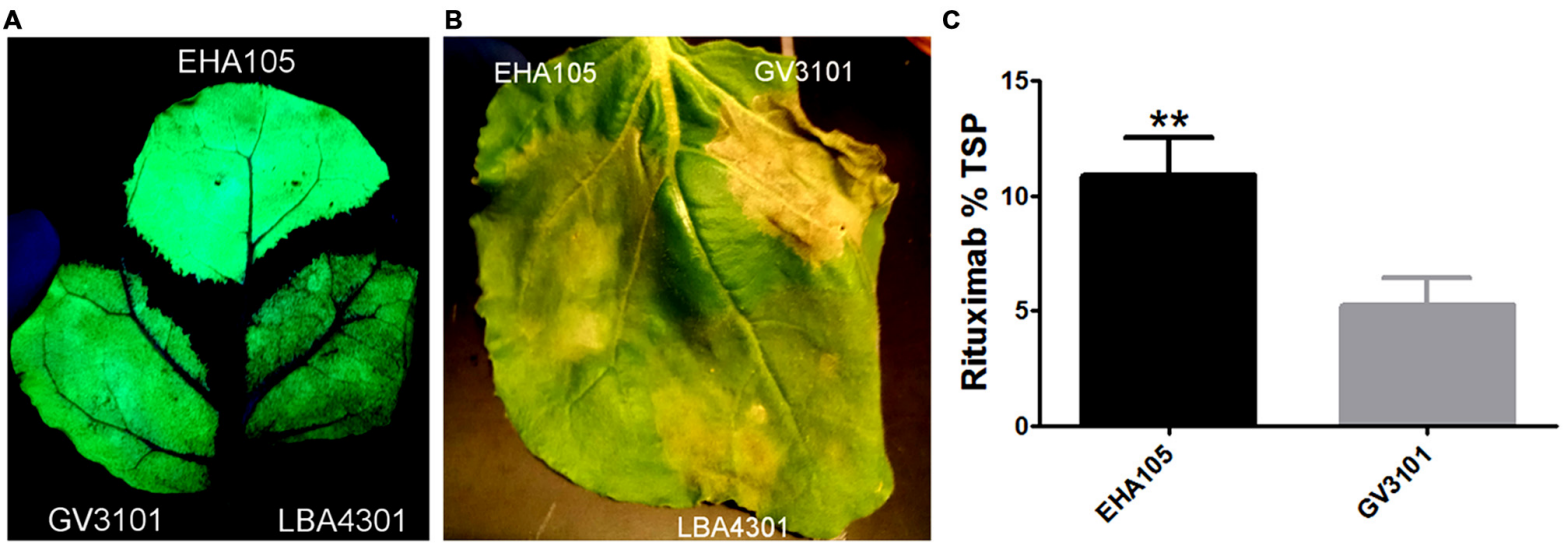
***Agrobacterium* strain EHA105 increases transgene production and reduces cell death. (A,B)** Leaves of *N. benthamiana* were infiltrated with *Agrobacterium* strains EHA105, GV3101, or LBA4301, each harboring a BeYDV GFP vector (pBYR2eFa-GFP). Representative images of four independently infiltrated leaves were photographed at 4 DPI under UV illumination **(A)** or 7 DPI under visible light **(B)**. **(C)** Leaves of *N. benthamiana* were infiltrated with *Agrobacterium* strains EHA105 or GV3101 harboring BeYDV rituximab vectors (pBYR2e-MRtxG and pBYR2e-MRtxK). Leaf extracts were analyzed for rituximab production by sandwich ELISA and data was normalized by total soluble protein. Columns represent data from four independently infiltrated samples ± standard error. Two stars (^∗∗^) indicates *p* < 0.05 by Student’s *t*-test.

The choice of *Agrobacterium* strain has been shown to play an important role in many aspects of transient protein production, including T-DNA transfer efficiency, plant health, and overall yield (Gleba et al., 2014; Shamloul et al., 2014; Sheikh et al., 2014). To investigate the effects of *Agrobacterium* strain on recombinant protein production, we introduced BeYDV GFP vectors to strains LBA4301, GV3101, and EHA105. Leaves of *N. benthamiana* were agroinfiltrated with each strain at OD600 of 0.2 and monitored for plant health and GFP production. At 4 DPI, spots infiltrated with GV3101 developed faint leaf browning, whereas the other two constructs had no detectable changes from uninfiltrated leaf tissue (data not shown). By 7 DPI, leaf spots infiltrated with GV3101 had become severely necrotic, while EHA105 or LBA4301 only had just begun to develop necrotic tissue (**Figure 7B**). Inspection of leaves under UV light revealed that fluorescing leaf regions infiltrated with EHA105 were substantially brighter than areas infiltrated with either of the other strains (**Figure 7A**).

To further compare the effects of EHA105 and GV3101 more quantitatively, BeYDV rituximab vectors were introduced to each strain and agroinfiltrated into *N. benthamiana* leaves. Leaf extracts were normalized for TSP and analyzed by IgG ELISA. In agreement with the data obtained using GFP vectors, *Agrobacterium* strain EHA105 substantially improved rituximab production: 10.9 ± 1.6 % TSP for EHA105 compared to 5.3 ± 1.1% TSP for GV3101 (**Figure 7C**). Additionally, the increase in rituximab production was correlated with a reduction in plant cell death. These results demonstrate the importance of *Agrobacterium* strain on improving recombinant protein production.

### Optimized Genetic Elements Function Synergistically to Further Enhance Transgene Production

**FIGURE 8:**
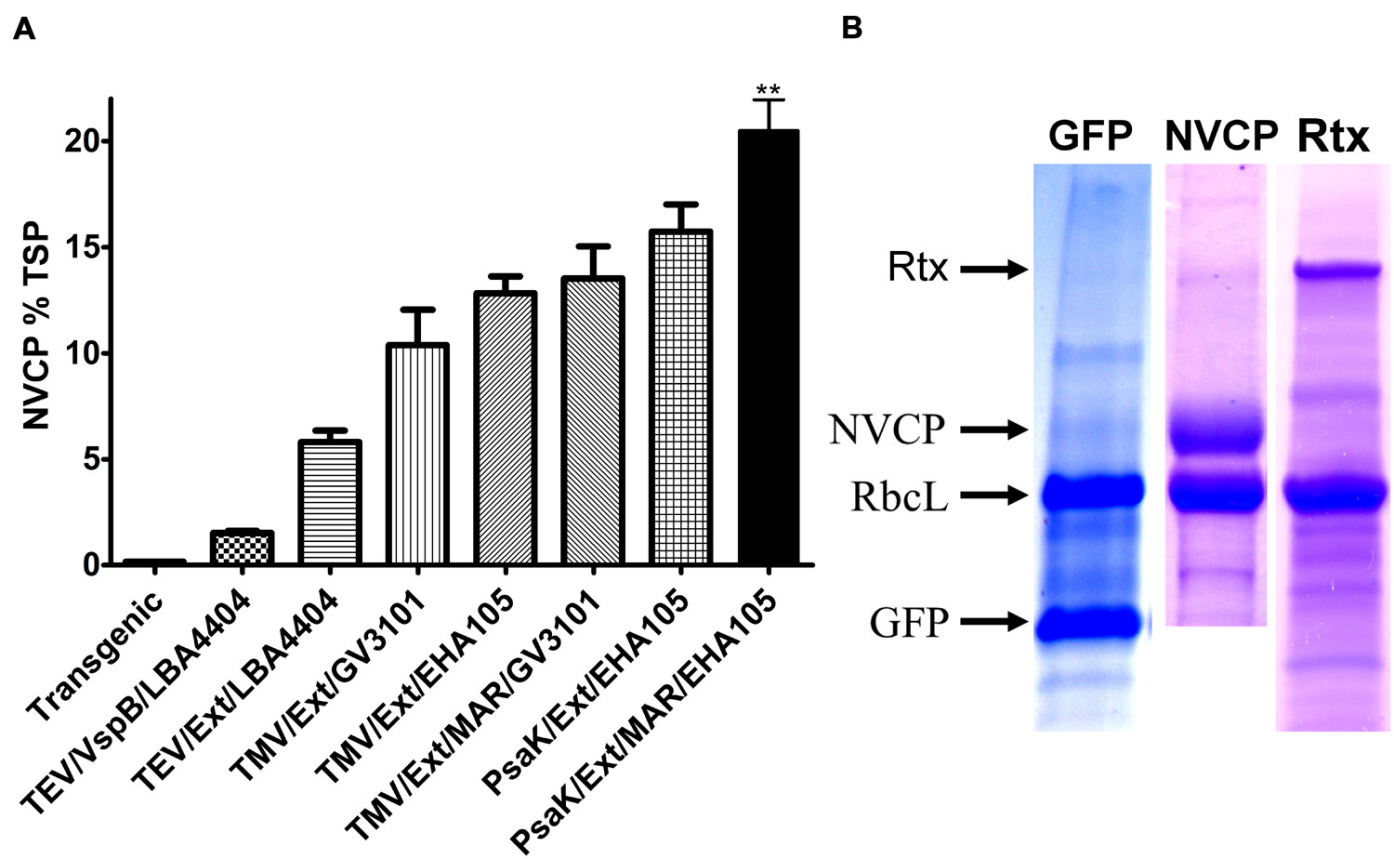
**BeYDV vector improvements enhance transgene production synergistically. (A)** Summary of improvements in NVCP production. Extracts from leaves of *N. benthamiana* agroinfiltrated with BeYDV NVCP vectors were analyzed by sandwich ELISA. Data were normalized for total soluble protein. Columns represent results from four or more independently infiltrated leaf samples ± standard error. Two stars (^∗∗^) indicates *p* < 0.05 as compared to AtPsaK/Ext/EHA105 by Student’s *t*-test. Transgenic, estimated yield (Santi et al., 2006); TEV, tobacco etch virus 5′ UTR; VspB, 3′ UTR from soybean *vspB* gene; LBA4404, *Agrobacterium* strain LBA4404; Ext, tobacco extensin terminator; TMV, tobacco mosaic virus 5′ UTR; GV3101, *Agrobacterium* strain GV3101; EHA105, *Agrobacterium* strain EHA105; MAR, tobacco Rb7 matrix attachment region inserted 3′ of the gene terminator; psaK, 5′ UTR from *A. thaliana* psaK gene. **(B)** Extracts from leaves of *N. benthamiana* agroinfiltrated using strain EHA105 with BeYDV GFP (pBYR2eP3-GFPM), NVCP (pBYR2eP3-sNVM), or rituximab vectors (Rtx, pBYR2ePSI-MrtxGM + pBYR2ePSI-MrtxKM) were separated on SDS-PAGE gels and stained with Coomassie dye. The bands corresponding to GFP (26 kDa) and NVCP (58 kDa) are indicated, along with the rubisco large subunit rbcL.

We determined the potential for the enhancing effects of the genetic elements identified in the present study to function synergistically with one another. *Agrobacterium* strain EHA105 was observed to perform better with all tested constructs, and was used for the remainder of studies (data not shown). BeYDV NVCP vectors were created which contained the AtPsaK 5′ UTR and extensin terminator with or without the 3′ Rb7 MAR, and were agroinfiltrated using strain EHA105 into *N. benthamiana* leaves. NVCP ELISA showed that insertion of the 3′ Rb7 MAR paired with the AtPsaK 5′ UTR and extensin terminator significantly enhanced NVCP production, yielding 20.3 ± 1.5% TSP compared to 15.7 ± 1.3% TSP for the construct lacking the MAR (**Figure 8A**, last two columns). This yield corresponds to 1.8 mg NVCP per gram leaf fresh weight. These results, compared with previous data, indicate that the optimizations identified in this study provide synergistic enhancement of transgene production, enabling very high levels of recombinant protein production (**Figures 8A,B**).

## Discussion

In recent years, transient expression systems have become the method of choice for plant-based recombinant protein production. In addition to their high yields, the rapid speed of these systems (4–5 days for BeYDV vectors) offers many unique advantages over stable transgenic systems, such as the ability to produce personalized therapeutics as reported for non-Hodgkin’s lymphoma (Bendandi et al., 2010; Tuse et al., 2015), and to rapidly respond to virus outbreaks or bioterrorism events (D’Aoust et al., 2010). Additionally, transient expression systems circumvent the regulatory issues associated with the creation of genetically modified organisms.

The most widely used transient expression system, magnICON, uses viral vectors derived from TMV and PVX (Giritch et al., 2006). Due to the competing nature of many RNA viruses, this system cannot produce recombinant proteins with more than two heterosubunits, excluding the efficient production of secretory IgAs, IgMs, and heteromultimeric virus-like particles, among other desirable biopharmaceuticals (Chen and Lai, 2013). A non-replicating system based on cowpea mosaic virus was has been used to produce bluetongue virus-like particles, allowing proper assembly of four heterosubunits (Thuenemann et al., 2013b). However, this system lacks the high yields associated with the other replicating systems (Gleba et al., 2014).

To circumvent these issues, we developed a transient expression system based on BeYDV which generates non-competing DNA replicons to drive high-level production of heteromultimeric proteins (Huang et al., 2010). While this system generates massive amounts of DNA copies of the target gene(s) which are thought to result in a saturation of the plant transcription machinery, the disparity between gene copy number, transcript accumulation, and protein production suggested that each transcript was not being efficiently utilized by the plant cell (Huang et al., 2009, 2010; Regnard et al., 2010). Therefore, we hypothesized that optimizing the genetic elements involved in efficient transcript processing, stability, and utilization could further improve the BeYDV system.

In the current study, we present a comprehensive comparison of diverse genetic elements and assess their potential to enhance plant-based recombinant protein production. We compared a large set of 5′ UTRs derived from human, plant, and viral sequences. In agreement with previous studies demonstrating cross-kingdom translational enhancement of certain 5′ UTRs (Dorokhov et al., 2002; Terenin et al., 2005), we found that many of the human sequences, as well as the polypurine 5′ UTR described by Dorokhov et al. (2002) provided high levels of GFP production in leaves of *N. benthamiana*, in some cases out-performing the routinely used viral 5′ UTRs from tobacco etch virus or AMV (**Figures 3A,B**). Among the virus-derived 5′ UTRs tested, we found the TMV 5′ UTR provided the highest level of transgene expression. Some of the viral elements tested, especially those containing long 3′ UTRs, performed very poorly. As RNA viruses are not typically adapted for the plant nucleus, many of these sequences may contain cryptic splice sites or other detrimental elements. We suspect that rigorous optimization of these sequences, such as through the insertion of introns and removal of sequences known to destabilize mRNA, could significantly improve the performance of genetic elements derived from these viruses.

Interestingly, despite the historic success of viral elements in driving high levels of protein production, we also found that the plant-derived 5′ UTRs from the psaK homologs of both *A. thaliana* and *N. benthamiana* were capable of enhancing recombinant production by as much as 40% more than the widely used TMV 5′ UTR (**Figure 4**). In particular, the first 40–60 nt directly upstream from the ATG seemed the most potent, possibly due to the removal of inhibitory regulatory sequences further upstream (**Figure 4**). Furthermore, we investigated the potential of the tobacco extensin terminator to enhance transgene production. It was found to prevent read-through transcription, enhance mRNA accumulation, and enhance protein production at a level greater than the 35S or nopaline synthase terminators, among other commonly used gene terminators (data to be presented elsewhere, **Figure 2**). We anticipate that further investigation of other native genetic elements from highly expressed plant genes has great potential to improve recombinant protein production systems.

Matrix attachment regions have a well-supported history of enhancing transgene production in transgenic plants (Halweg et al., 2005; Xue et al., 2005; Wang et al., 2007; Zhang et al., 2009; Ji et al., 2013), though their features seem variable or, in many cases, poorly understood. In the present study, we show that a MAR increases transgene production and reduces cell death in a plant transient expression system. Replicated geminivirus DNA has been shown to be organized into chromatin (Pilartz and Jeske, 1992; Pilartz and Jeske, 2003), and subject to repressive DNA methylation (Raja et al., 2008), indicating that MARs could be functionally active in BeYDV replicons. We found that insertion of the Rb7 MAR had a substantial enhancing effect on rituximab production, improving yield by 3.4-fold (**Figure 6A**). The MAR was most active when placed 3′ of the expression cassette, in contrast to other studies which found optimal placement upstream from the promoter, or in both positions (Zhang et al., 2009). Inspection of the tobacco Rb7 sequence reveals the presence of many polyadenylation and transcription termination signals, suggesting the alternative hypothesis that the 3′ MAR is acting as a second gene terminator or otherwise stabilizing the mRNA. Double terminators have been found to have a dramatic enhancing effect on transgene production (Beyene et al., 2011). Unexpectedly, we also found a dramatic decrease in cell death associated with the insertion of the tobacco Rb7 MAR (**Figure 6B**). Further studies are underway to characterize the function of the Rb7 MAR and other MARs in enhancing transgene production and reducing cell death in the BeYDV system.

One of the drawbacks of transient expression systems compared to stable transgenics is the requirement for *Agrobacterium* to deliver the gene of interest to the plants. An ideal *Agrobacterium* strain should minimize deleterious plant cell interactions while providing efficient T-DNA transfer to reduce the concentration of *Agrobacterium* required for complete gene delivery to all plant cells. We wished to evaluate different strains of *Agrobacterium* using the BeYDV system. EHA105 has been reported to overexpress *virG*, a transcriptional activator that regulates T-DNA transfer through induction of vir gene expression (Jin et al., 1987). Additionally, GV3101 and LBA4404 have been reported to have differing effects on the activation of plant immune response genes through the production of cytokinins (Sheikh et al., 2014). Previously, we found that strain GV3101 enhanced transgene production compared to strain LBA4404 (data not shown). In this study, we compared strains GV3101, LBA4301, and EHA105, and found that EHA105 both enhanced transgene production, and reduced plant cell death. Our results demonstrate that *Agrobacterium* strain can have a dramatic effect on recombinant protein production systems (**Figure 7**). A strain CryX is reported to provide 100–1000 times the gene delivery efficiency compared to commonly used *Agrobacterium* strains (Gleba et al., 2014). These studies indicate there is great potential to reduce plant toxicity and improve T-DNA transfer efficiency by optimizing the *Agrobacterium* strain.

## Conclusion

By optimizing the gene terminator, 5′ UTR, and *Agrobacterium* strain, and by targeted insertion of MAR elements, we have dramatically improved the BeYDV transient expression system. We have used this system to produce NVCP at up to 20% TSP, corresponding to 1.8 mg per gram leaf fresh weight, a >4-fold improvement over the original vector (Huang et al., 2009) and more than twice the highest level ever reported in a plant-based system (Santi et al., 2008). Furthermore, we have also produced the monoclonal antibody rituximab at up to 1 mg per gram leaf fresh weight, which is twice the highest level previously reported for a monoclonal antibody using BeYDV vectors (Huang et al., 2010). We expect these improvements to be broadly applicable to other DNA expression systems. Additionally, these modifications could be used to fine-tune expression in cases where multiple proteins need to be produced at different levels.

## Author Contributions

HM planned experiments, constructed expression vectors, and wrote and edited the ms. AD planned experiments, constructed expression vectors, performed experiments, and wrote the ms. SR planned experiments, constructed expression vectors, performed experiments, and wrote the ms.

## Conflict of Interest Statement

The authors declare that the research was conducted in the absence of any commercial or financial relationships that could be construed as a potential conflict of interest.

The reviewer GR and handling Editor declared their shared affiliation, and the handling Editor states that the process nevertheless met the standards of a fair and objective review.

## References

[B1] Agarwal P. Garg V. Gautam T. Pillai B. Kanoria S. Burma P. K. (2014). A study on the influence of different promoter and 5’UTR (URM) cassettes from *Arabidopsis thaliana* on the expression level of the reporter gene beta glucuronidase in tobacco and cotton. *Transgenic Res.* 23 351–363. 10.1007/s11248-013-9757-9 24072400

[B2] Becker D. Kemper E. Schell J. Masterson R. (1992). New plant binary vectors with selectable markers located proximal to the left T-DNA border. *Plant Mol. Biol.* 20 1195–1197. 10.1007/BF00028908 1463855

[B3] Bendandi M. S. Marillonnet R. Kandzia F. Thieme A. Nickstadt S. Herz R. (2010). Rapid, high-yield production in plants of individualized idiotype vaccines for non-Hodgkin’s lymphoma. *Ann. Oncol.* 21 2420–2427. 10.1093/annonc/mdq256 20494963

[B4] Beyene G. Buenrostro-Nava M. T. Damaj M. B. Gao S. J. Molina J. Mirkov T. E. (2011). Unprecedented enhancement of transient gene expression from minimal cassettes using a double terminator. *Plant Cell Rep.* 30 13–25. 10.1007/s00299-010-0936-3 20967448

[B5] Butler M. Meneses-Acosta A. (2012). Recent advances in technology supporting biopharmaceutical production from mammalian cells. *Appl. Microbiol. Biotechnol.* 96 885–894. 10.1007/s00253-012-4451-z 23053101 PMC7080107

[B6] Calikowski T. T. Meulia T. Meier I. (2003). A proteomic study of the *arabidopsis* nuclear matrix. *J. Cell. Biochem.* 90 361–378. 10.1002/jcb.10624 14505352

[B7] Carrington J. C. Freed D. D. (1990). Cap-independent enhancement of translation by a plant potyvirus 5’ nontranslated region. *J. Virol.* 64 1590–1597. 2319646 10.1128/jvi.64.4.1590-1597.1990 PMC249294

[B8] Chen Q. He J. Phoolcharoen W. Mason H. S. (2011). Geminiviral vectors based on bean yellow dwarf virus for production of vaccine antigens and monoclonal antibodies in plants. *Hum. Vaccin.* 7 331–338. 10.4161/hv.7.3.14262 21358270 PMC3166492

[B9] Chen Q. Lai H. (2013). Plant-derived virus-like particles as vaccines. *Hum. Vaccin. Immunother.* 9 26–49. 10.4161/hv.22218 22995837 PMC3667944

[B10] Chen Q. Lai H. (2015). Gene delivery into plant cells for recombinant protein production. *Biomed. Res. Int.* 2015:932161 10.1155/2015/932161 PMC4449920 26075275

[B11] Chiba Y. Green P. (2009). mRNA degradation machinery in plants. *J. Plant Biol.* 52 114–124. 10.1007/s12374-009-9021-2

[B12] D’Aoust M. A. Couture M. M. Charland N. Trepanier S. Landry N. Ors F. (2010). The production of hemagglutinin-based virus-like particles in plants: a rapid, efficient and safe response to pandemic influenza. *Plant Biotechnol. J.* 8 607–619. 10.1111/j.1467-7652.2009.00496.x 20199612

[B13] Ditt R. F. Nester E. W. Comai L. (2001). Plant gene expression response to *Agrobacterium tumefaciens*. *Proc. Natl. Acad. Sci. U.S.A.* 98 10954–10959. 10.1073/pnas.191383498 11535836 PMC58580

[B14] Dorokhov Y. L. Skulachev M. V. Ivanov P. A. Zvereva S. D. Tjulkina L. G. Merits A. (2002). Polypurine (A)-rich sequences promote cross-kingdom conservation of internal ribosome entry. *Proc. Natl. Acad. Sci. U.S.A.* 99 5301–5306. 10.1073/pnas.082107599 11959981 PMC122764

[B15] Fan Q. Treder K. Miller W. A. (2012). Untranslated regions of diverse plant viral RNAs vary greatly in translation enhancement efficiency. *BMC Biotechnol.* 12:22 10.1186/1472-6750-12-22 PMC3416697 22559081

[B16] Gallie D. R. Walbot V. (1992). Identification of the motifs within the tobacco mosaic virus 5′-leader responsible for enhancing translation. *Nucleic Acids Res.* 20 4631–4638. 10.1093/nar/20.17.4631 1408765 PMC334194

[B17] Gao R. Mukhopadhyay A. Fang F. Lynn D. G. (2006). Constitutive activation of two-component response regulators: characterization of VirG activation in *Agrobacterium tumefaciens*. *J. Bacteriol.* 188 5204–5211. 10.1128/JB.00387-06 16816192 PMC1539974

[B18] Gehrke L. Auron P. E. Quigley G. J. Rich A. Sonenberg N. (1983). 5′-Conformation of capped alfalfa mosaic virus ribonucleic acid 4 may reflect its independence of the cap structure or of cap-binding protein for efficient translation. *Biochemistry* 22 5157–5164. 10.1021/bi00291a015 6317016

[B19] Giritch A. Marillonnet S. Engler C. van Eldik G. Botterman J. Klimyuk V. (2006). Rapid high-yield expression of full-size IgG antibodies in plants coinfected with noncompeting viral vectors. *Proc. Natl. Acad. Sci. U.S.A.* 103 14701–14706. 10.1073/pnas.0606631103 16973752 PMC1566189

[B20] Gleba Y. Y. Tuse D. Giritch A. (2014). Plant viral vectors for delivery by Agrobacterium. *Curr. Top. Microbiol. Immunol.* 375 155–192. 10.1007/82_2013_352 23949286

[B21] Halweg C. Thompson W. F. Spiker S. (2005). The rb7 matrix attachment region increases the likelihood and magnitude of transgene expression in tobacco cells: a flow cytometric study. *Plant Cell* 17 418–429. 10.1105/tpc.104.028100 15659622 PMC548816

[B22] Huang Z. Chen Q. Hjelm B. Arntzen C. Mason H. (2009). A DNA replicon system for rapid high-level production of virus-like particles in plants. *Biotechnol. Bioeng.* 103 706–714. 10.1002/bit.22299 19309755 PMC2704498

[B23] Huang Z. Mason H. S. (2004). Conformational analysis of hepatitis B surface antigen fusions in an Agrobacterium-mediated transient expression system. *Plant Biotechnol. J.* 2 241–249. 10.1111/j.1467-7652.2004.00068.x 17147615

[B24] Huang Z. Phoolcharoen W. Lai H. Piensook K. Cardineau G. Zeitlin L. (2010). High-level rapid production of full-size monoclonal antibodies in plants by a single-vector DNA replicon system. *Biotechnol. Bioeng.* 106 9–17. 10.1002/bit.22652 20047189 PMC2905544

[B25] Jackson R. J. Hellen C. U. Pestova T. V. (2010). The mechanism of eukaryotic translation initiation and principles of its regulation. *Nat. Rev. Mol. Cell Biol.* 11 113–127. 10.1038/nrm2838 20094052 PMC4461372

[B26] Ji L. Xu R. Lu L. Zhang J. Yang G. Huang J. (2013). TM6, a novel nuclear matrix attachment region, enhances its flanking gene expression through influencing their chromatin structure. *Mol. Cells* 36 127–137. 10.1007/s10059-013-0092-z 23852133 PMC3887953

[B27] Jin S. G. Komari T. Gordon M. P. Nester E. W. (1987). Genes responsible for the supervirulence phenotype of *Agrobacterium tumefaciens* A281. *J. Bacteriol.* 169 4417–4425. 2443480 10.1128/jb.169.10.4417-4425.1987 PMC213802

[B28] Judge N. A. Mason H. S. O’Brien A. D. (2004). Plant cell-based intimin vaccine given orally to mice primed with intimin reduces time of *Escherichia coli* O157:H7 shedding in feces. *Infect. Immun.* 72 168–175. 10.1128/IAI.72.1.168-175.2004 14688094 PMC343997

[B29] Kanoria S. Burma P. K. (2012). A 28 nt long synthetic 5’UTR (synJ) as an enhancer of transgene expression in dicotyledonous plants. *BMC Biotechnol.* 12:85 10.1186/1472-6750-12-85 PMC3536603 23140609

[B30] Klimyuk V. Pogue G. Herz S. Butler J. Haydon H. (2014). Production of recombinant antigens and antibodies in *Nicotiana benthamiana* using ’magnifection’ technology: GMP-compliant facilities for small- and large-scale manufacturing. *Curr. Top. Microbiol. Immunol.* 375 127–154. 10.1007/82_2012_212 22527176

[B31] Lai H. He J. Engle M. Diamond M. S. Chen Q. (2012). Robust production of virus-like particles and monoclonal antibodies with geminiviral replicon vectors in lettuce. *Plant Biotechnol. J.* 10 95–104. 10.1111/j.1467-7652.2011.00649.x 21883868 PMC3232331

[B32] Liebich I. Bode J. Reuter I. Wingender E. (2002). Evaluation of sequence motifs found in scaffold/matrix-attached regions (S/MARs). *Nucleic Acids Res.* 30 3433–3442. 10.1093/nar/gkf446 12140328 PMC137072

[B33] Luo Z. Chen Z. (2007). Improperly terminated, unpolyadenylated mRNA of sense transgenes is targeted by RDR6-mediated RNA silencing in *Arabidopsis*. *Plant Cell* 19 943–958. 10.1105/tpc.106.045724 17384170 PMC1867362

[B34] Mason H. S. Ball J. M. Shi J. J. Jiang X. Estes M. K. Arntzen C. J. (1996). Expression of Norwalk virus capsid protein in transgenic tobacco and potato and its oral immunogenicity in mice. *Proc. Natl. Acad. Sci. U.S.A.* 93 5335–5340. 10.1073/pnas.93.11.5335 8643575 PMC39246

[B35] McCullen C. A. Binns A. N. (2006). *Agrobacterium tumefaciens* and plant cell interactions and activities required for interkingdom macromolecular transfer. *Annu. Rev. Cell Dev. Biol.* 22 101–127. 10.1146/annurev.cellbio.22.011105.102022 16709150

[B36] Mlynarova L. Hricova A. Loonen A. Nap J. P. (2003). The presence of a chromatin boundary appears to shield a transgene in tobacco from RNA silencing. *Plant Cell* 15 2203–2217. 10.1105/tpc.012070 12953121 PMC181341

[B37] Moore M. J. Proudfoot N. J. (2009). Pre-mRNA processing reaches back to transcription and ahead to translation. *Cell* 136 688–700. 10.1016/j.cell.2009.02.001 19239889

[B38] Mor T. S. Moon Y. S. Palmer K. E. Mason H. S. (2003). Geminivirus vectors for high-level expression of foreign proteins in plant cells. *Biotechnol. Bioeng.* 81 430–437. 10.1002/bit.10483 12491528

[B39] Mortimer C. L. Dugdale B. Dale J. L. (2015). Updates in inducible transgene expression using viral vectors: from transient to stable expression. *Curr. Opin. Biotechnol*. 32 85–92. 10.1016/j.copbio.2014.11.009 25437638

[B40] Nicolaisen M. Johansen E. Poulsen G. B. Borkhardt B. (1992). The 5′ untranslated region from pea seedborne mosaic potyvirus RNA as a translational enhancer in pea and tobacco protoplasts. *FEBS Lett.* 303 169–172. 10.1016/0014-5793(92)80511-E 1607015

[B41] Pilartz M. Jeske H. (1992). Abutilon mosaic geminivirus double-stranded DNA is packed into minichromosomes. *Virology* 189 800–802. 10.1016/0042-6822(92)90610-2 1641992

[B42] Pilartz M. Jeske H. (2003). Mapping of abutilon mosaic geminivirus minichromosomes. *J. Virol.* 77 10808–10818. 10.1128/JVI.77.20.10808-10818.2003 14512531 PMC224992

[B43] Raja P. Sanville B. C. Buchmann R. C. Bisaro D. M. (2008). Viral genome methylation as an epigenetic defense against geminiviruses. *J. Virol.* 82 8997–9007. 10.1128/JVI.00719-08 18596098 PMC2546898

[B44] Reavy B. Bagirova S. Chichkova N. V. Fedoseeva S. V. Kim S. H. Vartapetian A. B. (2007). Caspase-resistant VirD2 protein provides enhanced gene delivery and expression in plants. *Plant Cell Rep.* 26 1215–1219. 10.1007/s00299-007-0335-6 17370074

[B45] Regnard G. L. Halley-Stott R. P. Tanzer F. L. Hitzeroth I. I. Rybicki E. P. (2010). High level protein expression in plants through the use of a novel autonomously replicating geminivirus shuttle vector. *Plant Biotechnol. J.* 8 38–46. 10.1111/j.1467-7652.2009.00462.x 19929900

[B46] Richter L. J. Thanavala Y. Arntzen C. J. Mason H. S. (2000). Production of hepatitis B surface antigen in transgenic plants for oral immunization. *Nat. Biotechnol.* 18 1167–1171. 10.1038/81153 11062435

[B47] Sainsbury F. Thuenemann E. C. Lomonossoff G. P. (2009). pEAQ: versatile expression vectors for easy and quick transient expression of heterologous proteins in plants. *Plant Biotechnol. J.* 7 682–693. 10.1111/j.1467-7652.2009.00434.x 19627561

[B48] Santi L. Batchelor L. Huang Z. Hjelm B. Kilbourne J. Arntzen C. J. (2008). An efficient plant viral expression system generating orally immunogenic Norwalk virus-like particles. *Vaccine* 26 1846–1854. 10.1016/j.vaccine.2008.01.053 18325641 PMC2744496

[B49] Santi L. Huang Z. Mason H. (2006). Virus-like particles production in green plants. *Methods* 40 66–76. 10.1016/j.ymeth.2006.05.020 16997715 PMC2677071

[B50] Shamloul M. Trusa J. Mett V. Yusibov V. (2014). Optimization and utilization of Agrobacterium-mediated transient protein production in Nicotiana. *J. Vis. Exp.* 19 86 10.3791/51204 PMC4174718 24796351

[B51] Sheikh A. H. Raghuram B. Eschen-Lippold L. Scheel D. Lee J. Sinha A. K. (2014). Agroinfiltration by cytokinin-producing *Agrobacterium* sp. strain GV3101 primes defense responses in *Nicotiana tabacum*. *Mol. Plant Microbe Interact.* 27 1175–1185. 10.1094/MPMI-04-14-0114-R 25054409

[B52] Sugio T. Matsuura H. Matsui T. Matsunaga M. Nosho T. Kanaya S. (2010). Effect of the sequence context of the AUG initiation codon on the rate of translation in dicotyledonous and monocotyledonous plant cells. *J. Biosci. Bioeng.* 109 170–173. 10.1016/j.jbiosc.2009.07.009 20129102

[B53] Terenin I. M. Dmitriev S. E. Andreev D. E. Royall E. Belsham G. J. Roberts L. O. (2005). A cross-kingdom internal ribosome entry site reveals a simplified mode of internal ribosome entry. *Mol. Cell Biol.* 25 7879–7888. 10.1128/MCB.25.17.7879-7888.2005 16107731 PMC1190281

[B54] Thuenemann E. C. Lenzi P. Love A. J. Taliansky M. Becares M. Zuniga S. (2013a). The use of transient expression systems for the rapid production of virus-like particles in plants. *Curr. Pharm. Des.* 19 5564–5573. 10.2174/1381612811319310011 23394559

[B55] Thuenemann E. C. Meyers A. E. Verwey J. Rybicki E. P. Lomonossoff G. P. (2013b). A method for rapid production of heteromultimeric protein complexes in plants: assembly of protective bluetongue virus-like particles. *Plant biotechnol. J.* 11 839–846. 10.1111/pbi.12076 23647743

[B56] Tuse D. Ku N. Bendandi M. Becerra C. Collins R. Jr. Langford N. (2015). Clinical safety and immunogenicity of tumor-targeted, plant-made Id-KLH conjugate vaccines for follicular lymphoma. *Biomed. Res. Int.* 2015:648143 10.1155/2015/648143 PMC4575747 26425548

[B57] van der Fits L. Deakin E. A. Hoge J. H. Memelink J. (2000). The ternary transformation system: constitutive virG on a compatible plasmid dramatically increases Agrobacterium-mediated plant transformation. *Plant Mol. Biol.* 43 495–502. 10.1023/A:1006440221718 11052201

[B58] Veena Jiang H. Doerge R. W. Gelvin S. B. (2003). Transfer of T-DNA and Vir proteins to plant cells by *Agrobacterium tumefaciens* induces expression of host genes involved in mediating transformation and suppresses host defense gene expression. *Plant J.* 35 219–236. 10.1046/j.1365-313X.2003.01796.x 12848827

[B59] Wang T. Xue L. Hou W. Yang B. Chai Y. Ji X. (2007). Increased expression of transgene in stably transformed cells of *Dunaliella salina* by matrix attachment regions. *Appl. Microbiol. Biotechnol.* 76 651–657. 10.1007/s00253-007-1040-7 17611755

[B60] Wellensiek B. P. Larsen A. C. Stephens B. Kukurba K. Waern K. Briones N. (2013). Genome-wide profiling of human cap-independent translation-enhancing elements. *Nat. Methods* 10 747–750. 10.1038/nmeth.2522 23770754 PMC3731418

[B61] Xue H. Yang Y. T. Wu C. A. Yang G. D. Zhang M. M. Zheng C. C. (2005). TM2, a novel strong matrix attachment region isolated from tobacco, increases transgene expression in transgenic rice calli and plants. *Theor. Appl. Genet.* 110 620–627. 10.1007/s00122-004-1880-9 15660239

[B62] Zhang J. Lu L. Ji L. Yang G. Zheng C. (2009). Functional characterization of a tobacco matrix attachment region-mediated enhancement of transgene expression. *Transgenic Res.* 18 377–385. 10.1007/s11248-008-9230-3 19043795

[B63] Zhang X. Mason H. (2005). Bean Yellow Dwarf Virus replicons for high-level transgene expression in transgenic plants and cell cultures. *Biotechnol. Bioeng.* 93 271–279. 10.1002/bit.20695 16187337

